# Down-Regulation of DNA Mismatch Repair Enhances Initiation and Growth of Neuroblastoma and Brain Tumour Multicellular Spheroids

**DOI:** 10.1371/journal.pone.0028123

**Published:** 2011-12-01

**Authors:** Samuel L. Collins, Rodolphe Hervé, C. W. Keevil, Jeremy P. Blaydes, Jeremy S. Webb

**Affiliations:** 1 Centre for Biological Sciences, University of Southampton, Southampton, United Kingdom; 2 Environmental Health Care Unit, University of Southampton, Southampton, United Kingdom; 3 Southampton Cancer Research UK Centre, University of Southampton Faculty of Medicine, Southampton, United Kingdom; Enzo Life Sciences, Inc., United States of America

## Abstract

Multicellular tumour spheroid (MCTS) cultures are excellent model systems for simulating the development and microenvironmental conditions of *in vivo* tumour growth. Many documented cell lines can generate differentiated MCTS when cultured in suspension or in a non-adhesive environment. While physiological and biochemical properties of MCTS have been extensively characterized, insight into the events and conditions responsible for initiation of these structures is lacking. MCTS are formed by only a small subpopulation of cells during surface-associated growth but the processes responsible for this differentiation are poorly understood and have not been previously studied experimentally. Analysis of gene expression within spheroids has provided clues but to date it is not known if the observed differences are a cause or consequence of MCTS growth. One mechanism linked to tumourigenesis in a number of cancers is genetic instability arising from impaired DNA mismatch repair (MMR). This study aimed to determine the role of MMR in MCTS initiation and development. Using surface-associated N2a and CHLA-02-ATRT culture systems we have investigated the impact of impaired MMR on MCTS growth. Analysis of the DNA MMR genes *MLH1* and *PMS2* revealed both to be significantly down-regulated at the mRNA level compared with non-spheroid-forming cells. By using small interfering RNA (siRNA) against these genes we show that silencing of *MLH1* and *PMS2* enhances both MCTS initiation and subsequent expansion. This effect was prolonged over several passages following siRNA transfection. Down-regulation of DNA MMR can contribute to tumour initiation and progression in N2a and CHLA-02-ATRT MCTS models. Studies of surface-associated MCTS differentiation may have broader applications in studying events in the initiation of cancer foci.

## Introduction

First utilised by Sutherland et al [1]–[3] multicellular tumour spheroid (MCTS) cultures have become ideal model systems for studying many aspects of tumour growth and physiology [1]. Previous experimental approaches have shown that MCTS are morphologically and characteristically similar to solid tumours *in vivo* [1], [2], [4]. They are composed of symmetrically arranged aggregates of cells in discrete proliferating, dormant and necrotic populations and are subject to varying oxygen gradients, nutrient deficiencies and acidosis making them physiologically unique from standard 2-dimensional cultures. MCTS gene expression profiles also vary from 2-dimensional cultures and often closely mimic those of *in vivo* tumours [5], [6]. The ability to grow malignant cells as 3-D aggregates is of particular interest and to date spheroids have been used as important *in vitro* tumour model systems in several areas of cancer research [4].

Numerous properties of MCTS have been studied including metabolism [7], gene [5] and protein expression [8], growth kinetics [9]–[11], radiation resistance [12], [13] and drug therapy [14], [15]. There has been less insight, however, into the events and conditions that are responsible for the initiation of these structures. MCTS are formed by only a small subpopulation of cells during surface-associated growth. The mechanisms governing the differentiation between 2D culture and 3D MCTS are poorly understood and elucidation of these mechanisms may provide new insight into early events in tumourigenesis.

Tumour growth may be described as a process of cellular evolution [16] involving both genetic mutation and natural selection [17]. These processes are driven by numerous physiological and biochemical changes that occur during the progression of a healthy cell to a malignancy. Genetic mutations and associated genomic instability have received particular attention. Malignancy is characterised by the accumulation of a large number of mutations that cannot be accounted for by the low rate of spontaneous mutation typically found in somatic cells. This has led to the notion that cancer cells acquire a mutator phenotype early in malignant progression resulting from mutations in genes associated with maintaining genomic stability [18]. Genetic instability has been shown to play a crucial role in MCTS physiology, contributing, for example, to resistance to chemotherapeutic agents [5], [19] however its role in the early stages of spheroid formation is yet to be defined. One such mechanism for generating genetic instability is impaired DNA MMR. MMR mechanisms exist to remove nucleotide mismatches and insertion-deletion loops resulting from slippage of DNA polymerase during DNA replication. In human cells MMR is governed by several heterodimeric factors composed of at least six different proteins. MSH2 forms a heterodimer with either MSH6 or MSH3 (named MutSalpha and MutSbeta respectively). Both initiate the repair process, the former recognising base-base mispairs and the latter insertion-deletion loops. A heterodimer of *MLH1* and *PMS2* represents the major MutL activity in human cells and binds to the mismatch recognition complexes facilitating repair [20]–[22]. Previous evidence shows that inherited mutations of MMR genes are associated with high relative and absolute risk of cancer [23]–[26]. Hereditary nonpolyposis colon carcinoma (HNPCC) for example is associated with MMR deficiency and mutations in several of the human DNA MMR genes, prominently MSH2 and *MLH1* [24], [25], [27], [28].

Previous work by Francia *et al* [5], [19] revealed modest down-regulation of *PMS2* in EMT-6 mouse tumour spheroids. Subsequent work has shown partial down-regulation of *PMS2* in human cancer cell lines WM9, MDA-MB-231 and MDA-MB-435.TO.1 grown as spheroids compared with sub-confluent monolayer cultures and substantial down-regulation of *MLH1*, *PMS2* and, occasionally both genes, in human breast, prostate, and ovarian cancer cell lines [19]. Other components of the MMR system including MSH6 and MSH2 were also found to be differentially down-regulated in spheroid cultures. This observation is paralleled by an increase in the mutagenesis of reporter genes [29] and correlates well with the known physiology of MCTS. However, it remains to be understood if DNA MMR may contribute directly to the formation and growth of MCTS.

We have investigated the impact of down-regulated DNA MMR on the differentiation and progression of MCTS. In the present work, we have employed a murine neuroblastoma N2a and human brain tumour CHLA-02-ATRT surface-associated cell culture system and short inhibitory RNA (siRNA) gene silencing techniques to knockdown two critical MMR genes. Our data show that impaired MMR enhances MCTS initiation and progression, suggesting MMR mechanisms are involved in the processes that lead to the differentiation of MCTS during N2a and CHLA-02-ATRT surface-associated culture.

## Results

### Formation of multicellular tumour spheroids

**Figure 1:**
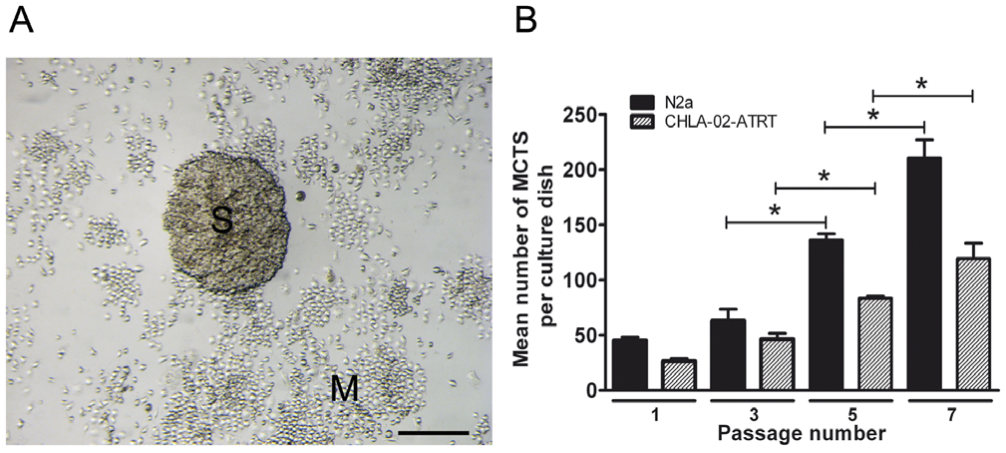
Subpopulations of murine N2a and human CHLA-02-ATRT cells form multicellular tumour spheroids during surface-associated culture. (A) Representative image of an N2a MCTS. N2a cells were plated at a density of 1×10^3^ cells per 60 mm dish and cultured under standard conditions for 5 days. Tumour spheroids (S) were differentiated from monolayer cells (M) at 6 days and exhibited a rough 3-dimensional appearance comparable to MCTS structures grown on non-adherent surfaces or in suspension. Scale bar = 100 µm. (B) MCTS production increased with serial passage. N2a and CHLA-02-ATRT cells were plated at a density of 1×10^3^ cells per 60 mm dish and cultured for 6 days. MCTS number was recorded. Cells were scraped from the dish surface, treated with non-enzymatic cell dissociation solution and strained over a 70 µm filter to produce a single-celled suspension which was verified with a haemocytometer. New 60 mm dishes were plated with 1×10^3^ cells from this suspension and cultured for a further period of 6 days. MCTS number was again recorded. This procedure was repeated for a total of 7 passages. Statistical differences were calculated by Student's *t*-test. Each bar represents the mean ± s.e.m. for at least three independent experiments. Significant differences (P<0.001) are indicated by asterisks (*).

Murine neuroblastoma N2a and human CHLA-02-ATRT (intracranial atypical rhabdoid tumour) cells spontaneously formed MCTS when grown on adhesive tissue culture plastic. Spheroids displayed a broad size distribution ranging from 30–600 µm in diameter (Fig. 1A). Both cell lines exhibited an enhanced ability to form spheroids over successive passages (Fig. 1B) with an approximate quadrupling of spheroid number between passage 1 and 7 for N2a cells (P<0.004). Spheroids were not observed to coalesce when dense. Live video microscopy indicated that spheroids were clonal in nature, showing internal expansion of spheroid structure as opposed to external aggregation of motile cells associated with the plate surface.

### Down-regulation of *MLH1* and *PMS2* in MCTS

**Figure 2:**
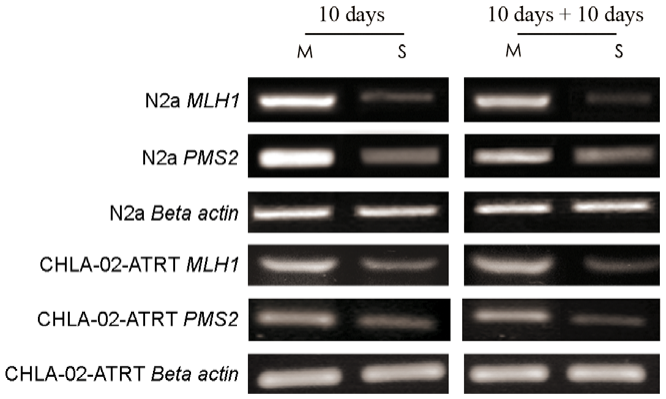
Decreased expression of *MLH1* and *PMS2* in multicellular tumour spheroids. N2a and CHLA-02-ATRT cells were grown for 10 days in 90 mm dishes to generate large numbers of MCTS. MCTS were separated from the bulk monolayer culture using a 19 gauge needle. Spheroids were dissociated using non-enzymatic cell dissociation solution and strained over a 40 µm filter to produce a single-celled suspension. The remaining monolayer cells were scraped from the plate surface and treated as above to produce a single-celled suspension. Half of each of the spheroid and monolayer-derived cells were lysed and total RNA extracted from both cell populations. Reverse transcription PCR was conducted to analyse differences in *MLH1* and *PMS2* gene expression between the dissociated spheroid (S) and non-spheroid forming monolayer cells (M). A reduced expression of both *MLH1* and *PMS2* was detected in spheroid cells compared to the bulk monolayer cells. The remaining dissociated spheroid cells and non-spheroid forming monolayer cells were re-seeded as separate populations in new dishes and grown for a further 10 days. RT-PCR was performed on RNA extracted from the entire cell population and analysis revealed a similar decreased expression of *MLH1* and *PMS2* in spheroid-derived cells compared to monolayer-derived cells. Beta actin expression is shown to confirm equal sample loading.

To determine whether the MMR genes *MLH1* and *PMS2* are differentially expressed in N2a and CHLA-02-ATRT MCTS we carried out reverse transcription PCR (RT-PCR) analyses on separated monolayer and spheroid-derived cells. Analysis revealed marked down-regulation *MLH1* and *PMS2* expression in MCTS compared with cells derived from the ‘non-spheroid-forming’ 2D monolayer. Our data shows a moderate down-regulation of both *MLH1* and *PMS2* in 10 day old spheroids compared with monolayer cells (Fig. 2). A similar decreased expression of *MLH1* and *PMS2* was detected in spheroid cells that had been dissociated, re-seeded and cultured for a subsequent 10 day period. The down-regulation of *MLH1* and *PMS2* observed in these experiments is comparable to previous work conducted on different murine tumour cell lines grown as spheroids [5], [19].

### Impact of MMR gene silencing on MCTS initiation and development

**Figure 3:**
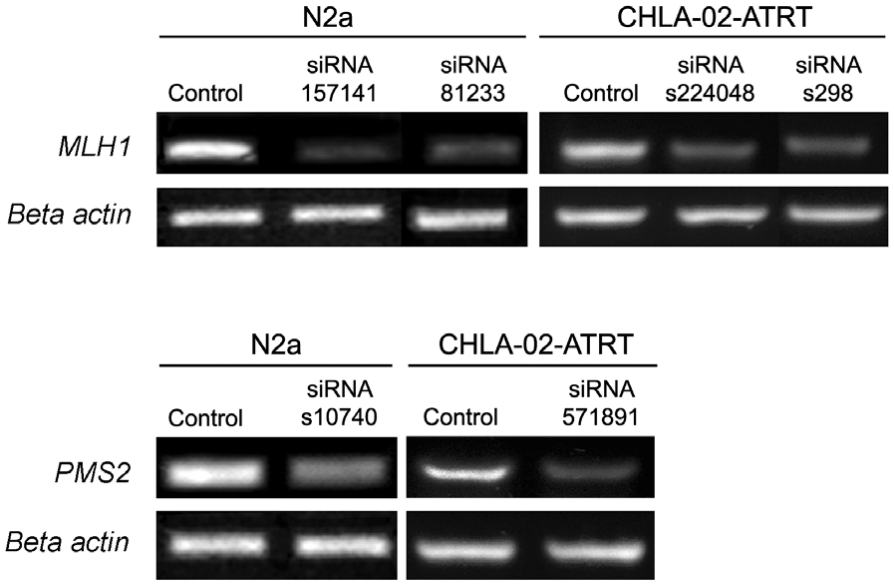
Confirmation of siRNA silencing activity. N2a and CLHA-02-ATRT cells at 50% confluence were transfected with 30 nM siRNA targeted against *MLH1* and *PMS2* and a negative control scrambled siRNA for a period of 5 hours. At 72 hours post-transfection cells were lysed and total RNA extracted. Reverse transcription PCR analyses were conducted and confirmed successful knockdown of *MLH1* and *PMS2* expression in both cell types following siRNA transfection. Beta actin expression is shown for both the control and targeted siRNA treated cells to confirm equal sample loading.

**Figure 4:**
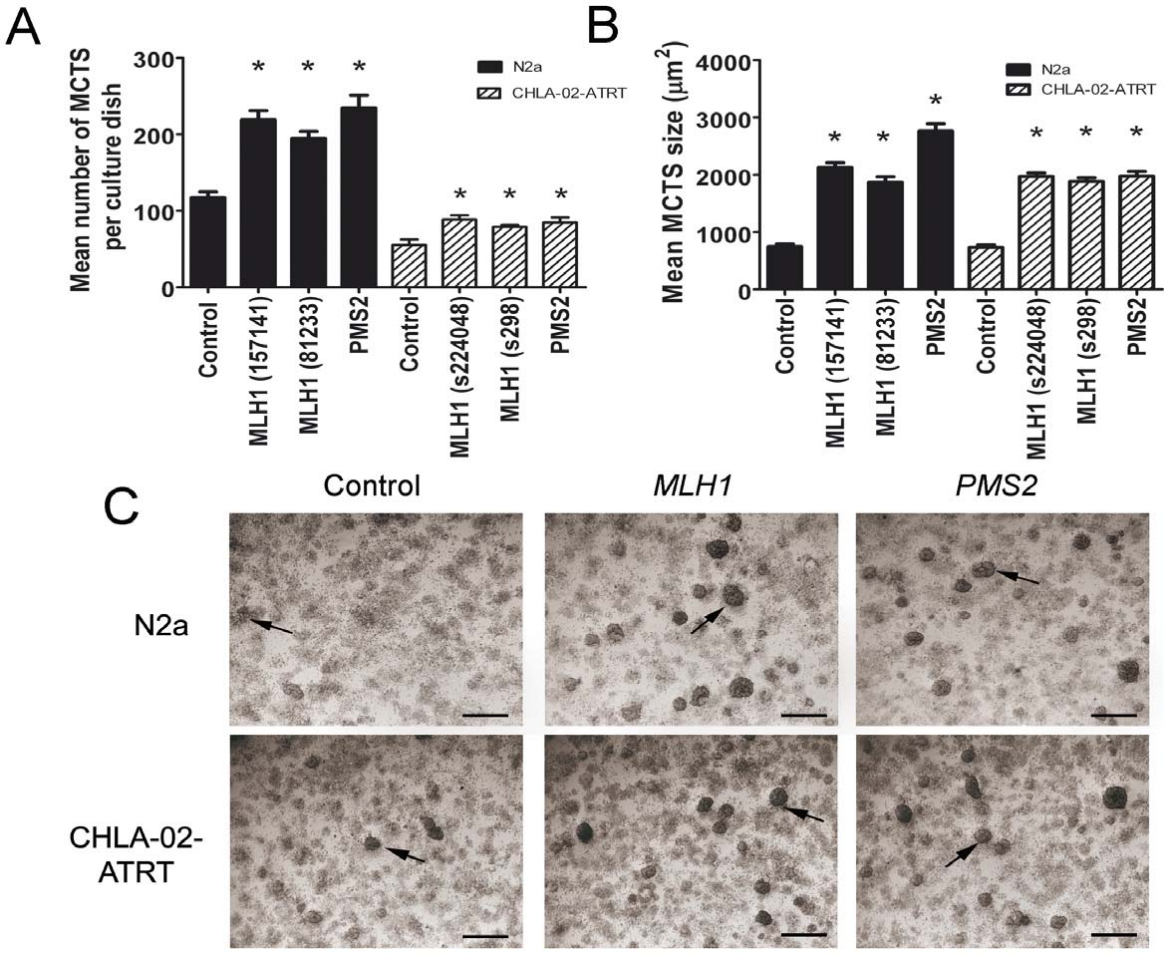
Silencing of *MLH1* and *PMS2* results in enhanced MCTS formation. N2a and CHLA-02-ATRT cells at 50% confluence were transfected with 30 nM siRNA targeted against *MLH1* and *PMS2* and a negative control scrambled siRNA for a period of 5 hours. Cultures were incubated under standard conditions for a further period of 4 days. MCTS number and size were determined by light microscopy and image analysis. The silencing of *MLH1* and *PMS2* resulted in a significant increase in both the number (A) and size (B) of MCTS for both N2a and CHLA-02-ATRT cell lines. Statistical differences were calculated by One Way ANOVA between the negative control and *MLH1*/*PMS2* targeted siRNA cultures. Each bar represents the mean +/− s.e.m. for at least three independent experiments. Significant differences (P<0.05) are indicated by the asterisks (*). (C) Representative low magnification light microscopy images showing cultures of N2a and CHLA-02-ATRT cells transfected with 30 nM negative control, *MLH1* and *PMS2* targeted siRNA. MCTS appear as dense 3-dimensional structures as indicated by the arrows. Images were obtained 4 days post-transfection. Scale bar represents 200 µm.

To investigate the effect of impaired DNA MMR on the initiation and progression of N2a and CHLA-02-ATRT MCTS we employed a siRNA silencing technique to knockdown the expression of the MMR genes *MLH1* and *PMS2*. Gene expression knockdown was confirmed by RT-PCR (Fig. 3). Spheroid number and size was determined 7 days post-transfection using low magnification microscopy and image analysis software. Cultures transfected with *MLH1* and *PMS2* siRNA exhibited enhanced MCTS formation with a mean 225±23.1 and 242±35 spheroids per 60 mm plate respectively for N2a, compared to control siRNA transfected cells of 133±17.3 and a mean 88.5±5.6 and 84.7±6.6 respectively for CHLA-02-ATRT compared to control siRNA transfected cells of 55.4±7.1 (Fig. 4A). We also observed an approximate 175% and 168% increase in the size of spheroids formed by transfected N2a and CHLA-02-ATRT cultures respectively (Fig. 4B). We detected no increase in spheroid number or size with cultures incubated with scrambled negative control siRNA or with the transfection agent only (data not shown).

Transfection experiments were repeated on the non-tumour murine fibroblast cell line 3T3. We observed no spontaneous formation of differentiated MCTS in control or transfected cultures (data not shown).

**Figure 5:**
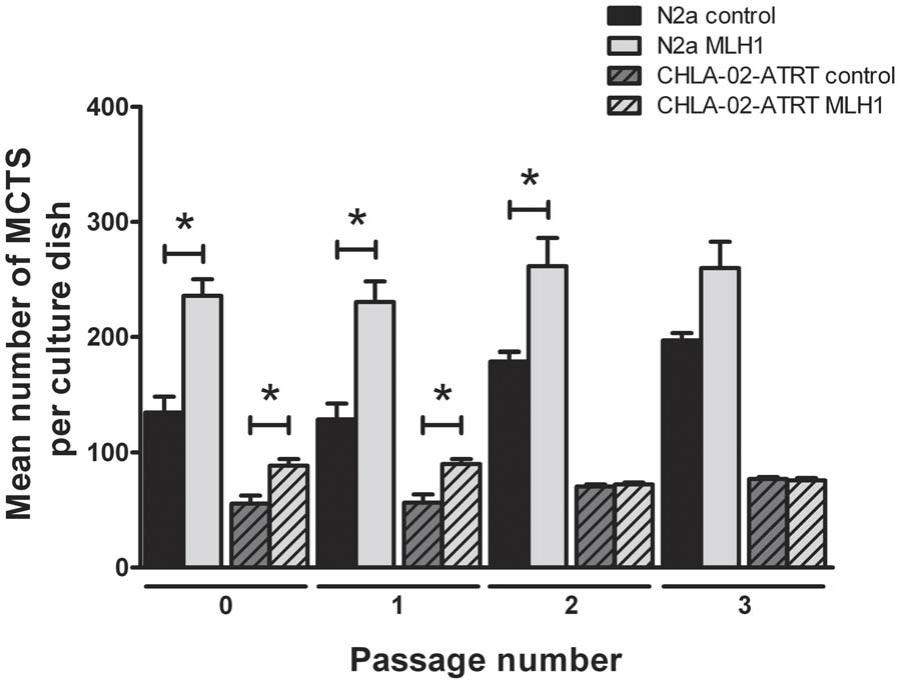
N2a and CHLA-02-ATRT cultures transfected with *MLH1* siRNA continue to form higher numbers of MCTS following post-transfection passage. Negative control and *MLH1* siRNA transfected cultures were grown for 4 days post-transfection and analysed for MCTS formation (passage number 0). Control and *MLH1* transfected cultures were then scraped from the plate surface, treated with non-enzymatic cell-dissociation, strained over a 70 µm filter to produce a single cell suspension and re-seeded as separate populations in new 60 mm dishes at a density of 1×10^3^ cells per dish. MCTS number was analysed 7 days post passage. This procedure was repeated for a total of 3 passages. N2a *MLH1* siRNA transfected cultures were seen to retain an increased MCTS forming ability even after 3 passages. CHLA-02-ATRT *MLH1* transfected cultures showed an increased propensity to form higher numbers of MCTS for one post-transfection passage only. Each bar represents the mean +/− s.e.m. for at least three independent experiments. Significant differences (P<0.05) are indicated by the asterisks (*) and were calculated using the Student's *t*-test.

In order to assess the duration of the effects induced by *MLH1* silencing, transfected cultures were passaged at day 7 and reseeded into new tissue culture plates. MCTS analysis was conducted 7 days thereafter and the cultures passaged once again. This procedure was repeated for a total of 3 post-transfection passages. N2a *MLH1* siRNA transfected cultures were seen to retain an statistically significant increased MCTS forming ability after 2 post-transfection passages, whereas CHLA-02-ATRT *MLH1* transfected cultures showed a significant increase in MCT formation for one post-transfection passage only (P<0.05) (Figure 5). MCTS numbers in both control and transfected N2a cultures were seen to increase with passage number – consistent with previous findings outlined in this paper.

### Spheroid-derived cells exhibit enhanced MCTS growth

**Figure 6:**
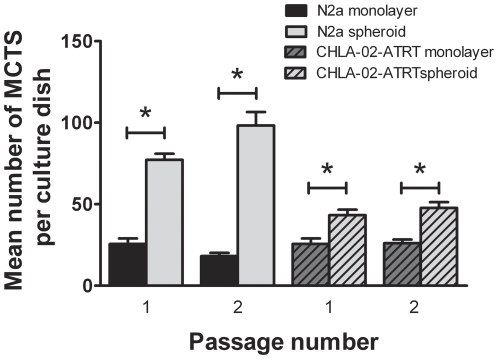
Spheroid-derived cells show an increased popensity to form new spheroids. N2a and CHLA-02-ATRT MCTS were separated from 7 day old cultures using the method described previously in this paper. The MCTS and remaining non-spheroid-forming monolayer cells were treated with non-enzymatic cell dissociation solution to produce single cell suspensions and re-seeded as separate populations into new 60 mm dishes at a density of 1×10^3^ cells per dish. Spheroid-derived cells exhibited a higher propensity to form MCTS compared to monolayer-derived cells. Each bar represents the mean +/− s.e.m. for at least three independent experiments. Significant differences (P<0.05) are indicated by the asterisks (*) and were calculated using the Student's *t*-test.

To assess whether MCTS cells undergo genetic or epigenetic changes that enhance their ability to form MCTS we separated spheroids from 7 day old cultures, dissociated them using non-enzymatic cell dissociation solution and plated them as single cell cultures onto new adhesive tissue culture plates. Non-spheroid forming monolayer cells from the same cultures were plated at equal density on separate plates. Spheroid-derived cells exhibited a higher propensity to form new MCTS compared to non-spheroid monolayer cells (Fig. 6).

## Discussion

The work presented here is the first to causally link down-regulated DNA MMR gene expression with the initiation and development of MCTS *in-vitro*. We used silencing RNA (siRNA) to knockdown expression of the MMR genes *MLH1* and *PMS2* in surface-associated N2a and CHLA-02-ATRT cultures. Our data show that silencing of these two genes results in an increase in the number and size of MCTS that was observed over several subsequent passages following transfection. The use of both murine and human tumour cell lines eliminates the possibility of the observations being species-specific.

Several possibilities exist to explain why impaired DNA MMR enhances MCTS growth. MMR proteins serve to correct post-replication errors by eliminating base-base mismatches and insertion or deletion loops. An increase in spontaneous mutation frequency resulting from an impairment of this system may give rise to a range of beneficial and deleterious mutations. Such a mechanism has been demonstrated by Nicolaides *et al* [30] who, in an alternative approach to siRNA performed transfection studies using a naturally occurring mutant h*PMS2* identified from the germline of a HNPCC patient. The nonsense mutation was sufficient to suppress DNA MMR pathways and induce microsatellite instability in cells containing wild-type h*PMS2*. Mutator phenotypes of this kind represent an important model for cancer development whereby mutant cells selected for their ability to proliferate, while surviving environmental stresses, expand their numbers and contribute to MCTS growth [18], [31]. This hypothesis is supported by data in this paper which reports that spheroid-derived cells exhibit a higher propensity to form MCTS compared to cells derived from the 2-D monolayer

However, MMR proteins have several functions other than direct DNA repair that are relevant to genomic stability and carcinogenesis. These include DNA damage surveillance, damage signaling and apoptosis [24], prevention of recombination between non-identical sequences [32], [33] and participation in meiotic processes (chromosome pairing) [33]. In addition there is now evidence that the MMR system is integral to the signaling processes that activate cell-cycle checkpoints or apoptosis. Following DNA damage, cell cycle checkpoint activation halts the cell cycle allowing time for DNA repair. Repair occurs in the G1-S and G2-M phases. Previous studies have shown that defective expression of *MLH1* alters G2-M cell cycle checkpoint control in human colon carcinoma cells exposed to ionizing radiation [34]. Similar observations have been made in *MLH1* deficient mice. Further work has suggested that MMR complexes play a key role in linking Ataxia telangiectasia mutated (ATM) and the downstream effector, CHK2 checkpoint homolog (CHEK2), in response to DNA damage. CHK2 activation is lost in cells deficient in MMR causing the G1-S checkpoint to fail. Such interactions suggest a relationship between checkpoint signaling activated by DNA damage and mechanisms of DNA repair. In addition to the mutator phenotype associated with deficiency in MMR, abrogated S-phase checkpoint activation may further explain the genomic instability and cancer predisposition arising from inactivation of the MMR system.

It must also be stated that the MMR system can mediate cytotoxicity. Down-regulation of MMR has been directly implicated in the resistance of cancers to radiation and a number of chemotherapeutic agents [35]. Impaired MMR results in drug resistance through two mechanisms: the inability to detect DNA damage and subsequent activation of apoptosis and through and increase in mutation rate throughout the genome [36]. Further studies to explore the clinical significance of MMR deficient cells in tumours are needed.

Previous data has also suggested that human neuroblastoma cell lines contain pluripotent tumour-initiating cells or ‘cancer stem cells’ distinguished by CD133 (a neural stem cell marker) expression [37]. Cancer stem cells have been seen to comprise the majority of a tumour mass before terminal differentiation [38]. Current models of cancer development imply that genetic instability and thus mutations directly affect normal stem cells and their cellular pathways causing them to become malignant [39]–[44]. In neuroblastoma CD133 positive cells show enhanced MCTS formation [37]. It is therefore possible that down-regulated DNA MMR may contribute to the genetic instability in stem cells, promoting MCTS initiation and growth.

Consistent with reports on numerous other human and murine cell lines grown as spheroids [5], [19] we have demonstrated down-regulation of *MLH1* and *PMS2* expression in N2a and CHLA-02-ATRT MCTS compared with non-spheroid forming ‘monolayer cells’. Recent evidence indicates that the expression of key DNA MMR genes is down-regulated under conditions of hypoxic stress. Hypoxia is a common feature of the tumour microenvironment, arising from imbalances between oxygen supply and consumption. Emerging evidence suggests that hypoxia-induced genetic instability is a key mechanism underlying tumour propagation. Reynolds *et al.* [45] and more recently Papp-Szabo *et al.* [46] have reported increased mutation frequencies in cells exposed to hypoxia-reoxygenation cycles. Further studies have implicated hypoxia in oxidative base damage [47], [48], gene amplification [49] and DNA over-replication [50]. Importantly for this present study hypoxia has been implicated in the repression of nucleotide MMR pathways and *MLH1* down-regulation is specifically enhanced under conditions of hypoxic stress [51]. Koshiji *et al* [52] have since demonstrated the down-regulation of MSH2 and MSH6 in response to hypoxia. Further alterations in the expression and activation patterns of numerous DNA repair and stress-response factors have been detected, however, the mechanisms responsible for these observations remains to be determined. Further work in our laboratory will examine the interrelationship between hypoxia and genetic instability in the context of MCTS initiation and development.

In summary our results show a deficiency of DNA MMR gene expression in murine N2a and human CHLA-02-ATRT cells grown as MCTS in comparison with 2D monolayer cultures. We have shown that down-regulated DNA MMR results in enhanced MCTS initiation and growth in N2a and CHLA-02-ATRT surface-associated culture systems. This shows that down-regulation of MMR can initiate and perpetuate the spheroid microenvironment, which can be an important initiating event in malignancy. Additional work such as an *in vitro* DNA repair assay (comparing the ability of cytosolic extracts to repair DNA mismatches [53]) could functionally assess the level of DNA mismatch repair activity in tumour spheroids or in the siRNA transfected cells and provide a clearer understanding of the processes that might be responsible for these findings. We believe that by examining distinct MCTS and monolayer subpopulations that arise during surface-culture of N2a and CHLA-02-ATRT, this study has addressed pathways that lead to the initiation and early differentiation of MCTS structures, thus providing new insight into early events in tumourigenesis.

## Materials and Methods

### Cell and spheroid culture

Use of neuroblastoma and brain tumour cell lines for studies of MCTS is well documented within the literature [5], [19], [37], [54], [55] and the murine neuro-2a (N2a) and human CHLA-02-ATRT (intracranial atypical rhabdoid tumour) cell lines readily form MCTS in our experimental system. The C_1300_ mouse N2a cell line [56] was obtained from the American Type Culture Collection (Manassas, VA, USA) at passage number 182. Any reference to passage number in the text refers to this number plus the given ‘P’ number. This cell line was maintained in minimal essential medium (MEM) (Invitrogen, Paisley, United Kingdom) supplemented with 10% foetal bovine serum (FBS) (Biosera, Ringmere, United Kingdom), 10% penicillin-streptomycin mix and 10% glutamine at 37°C and in an humidified atmosphere of 5% CO_2_. CHLA-02-ATRT cells were obtained from the ATCC at passage number 7. Any reference to the passage number in the text refers to this number plus the given ‘P’ number. This cell line was maintained in DMEM:F12 medium supplemented with 10% foetal bovine serum, 10% penicillin-streptomycin mix, 20 ng/mL human recombinant EGF, 20 ng/mL human recombinant basic FGF (R&D Systems, Abingdon, United Kingdom) and 2% B-27 supplement (Invitrogen). Murine 3T3 cells were maintained in MEM supplemented with 10% foetal bovine serum, 10% penicillin-streptomycin mix and 10% glutamine. Media were replaced every 3 days until cells had achieved 70% confluence and then every 2 days thereafter. At approximately 85% confluence, cell cultures were passaged by washing with sterile 1× Dulbecco's Phosphate Buffered Saline (D-PBS) without calcium and without magnesium and treating with TrypLE Express solution (Invitrogen) for 5 minutes at 37°C and 5% CO_2_. The resulting cell suspension was pelleted, the supernatant removed and cells resuspended in fresh warmed medium before reseeding in T75 flasks (Nunc, Roskilde, Germany) at a final concentration of 2×10^5^/ml.

MCTS were generated by seeding 1×10^3^ cells/plate on Nunclon Δ Surface coated 60 mm tissue culture plates (Nunc). Spheroids formed spontaneously over a period of 3–7 days. Media were changed according to cell confluence as previously described. Where indicated 2×10^4^ cells/well were plated on 90 mm pre-coated plates (Nunc) to scale up the experiment.

### Removal and dissociation of spheroids

Individual spheroids were ‘picked’ from the plate surface using a 19-gauge Microlance needle under a Leica MZ16F dissection microscope and transferred to pre-warmed appropriate media. Spheroids were dissociated into single cells using non-enzymatic dissociation solution (Sigma, Poole, United Kingdom) and strained over a 40 µm filter. The remaining monolayer cells were trypsinised from the plate surface and transferred to pre-warmed media. Both suspensions were pelleted at 3000 rpm for 3 minutes, and resuspended in fresh pre-warmed media. Culture plates were then seeded with 1×10^3^ spheroid or monolayer derived cells per plate and incubated at 37°C and 5% CO_2_. Media were changed according to cell confluence. Spheroid formation was analysed after 7–10 days.

### Image analysis and spheroid quantification

A Leica dissection microscope with attached Canon Powershot A470 digital camera was used to image and quantify all spheroids in 60 mm and 90 mm plates. For higher magnification images a Nikon Diaphot inverted microscope was used with attached Nikon D50 digital camera. Spheroid number and size was determined through image analysis using the NIH ImageJ software package.

### siRNA transfection

For RNA interference siRNA molecules were purchased from Ambion (Warrington, United Kingdom), two targeted against murine *MLH1* (157141 and 81233), one against murine *PMS2* (571891), two against human *MLH1* (s224048 and S298), one against human *PMS2* (s10740) and a negative control scrambled siRNA. Unless otherwise indicated all transfections were performed with a final concentration of 30 nM siRNA.

N2a and CHLA-02-ATRT cells were seeded at 1×10^3^ per plate into pre-coated 60 mm plates in a total volume of 4 mL MEM and incubated for 2–4 days until 50% confluent. Transfections were performed using INTERFERin™ transfection reagent (Polyplus, New York, USA) as per the manufacturer's instructions. Briefly siRNA complexes were prepared as aqueous solutions by adding the appropriate volume of siRNA to 200 µl warmed serum-free MEM media. 10 µl INTERFERin™ was added to each preparation and solutions incubated at room temperature for 10 minutes. Media were replaced with fresh MEM without antibiotics to which was added the siRNA-INTERFERin™ complexes to a total volume of 2 ml. Media containing the transfection agent was aspirated after 5 hours and replaced with fresh antibiotic-containing media.

### RNA isolation and cDNA synthesis

Total RNA was isolated using an RNeasy Mini kit (Qiagen, Crawley, United Kingdom) as described in the manufacturer's instructions. The extracted RNA was then used immediately or stored at −80°C until required. One µg of total RNA from each experimental set was prepared using a Nanodrop ND1000 machine (Thermo Scientific, Wilmington, USA) and used for cDNA synthesis. Reverse transcription of isolated RNA was achieved using the ImProm-II Reverse Transcription System (Promega, Southampton, United Kingdom) and oligo-dT primers.

### PCR and analysis of gene knockdown

cDNA were amplified by PCR using the GoTaq Green Master Mix kit (Promega) with specific primers for the amplification of murine and human *MLH1* and *PMS2*.

Sequences were as follows: Murine *MLH1* sense 5′-AGCAGCACATTGAGAGCAAG-3′, antisense 5′-GCTTACAGGCTGCAGAAAGG-3′ (224-bp product), murine *PMS2* sense 5′-CCAAGTGAGAAAAGGGGCGTGTTATCC-3′, antisense 5′-CTGTCTTGAAGCGCTTGGCATTTGTG-3′ (394-bp product), human *MLH1* sense 5′-GAGACAGTGGTGAACCGCAT-3′, antisense 5′-CTTGATTGCCAGCACATGGT-3′ (403-bp product) and human *PMS2* sense 5′-AGAACCTGCTAAGGCCATCA-3′, antisense 5′-TAAGCCTTCGAAGTTTTCTTCTT-3′ (233-bp product) [51]. For *β-actin*, the Mouse/Rat β-Actin primer pair (RnD Systems, Abingdon, UK) was used (302-bp product). PCR was conducted using an MJ Mini thermocycler (BioRad, Hemel Hempstead, United Kingdom).

PCR products were visualised by gel electrophoresis using a 1.5% agarose gel. Image acquisition of detected bands was carried out using a Genius Bio Imaging System (Syngene, Cambridge, United Kingdom).

### Statistical Analysis

All data were expressed as means ± s.e.m. for at least three independent experiments and analysed by One Way ANOVA or the Student's *t*-test Significant differences are indicated in the figures by an asterisk. SigmaStat 3.5 software was used to perform statistical tests.
